# Relationship between BMI-SDS trajectories and childhood asthma: a prospective cohort study from the Japan Environment and Children’s Study.

**DOI:** 10.1265/ehpm.26-00162

**Published:** 2026-09-29

**Authors:** Sachika Akaike, Toshihide Sakuragi, Kazue Ishitsuka, Reiko Suga, Keisuke Shimizu, Keiko Shimizu, Honoka Tomioka, Kazuhiro Yatera, Kiyoshi Yoshino, Reiji Fukano, Yuko Mine, Masayuki Ochiai, Seiichi Morokuma, Norio Hamada, Mayumi Tsuji, the Japan Environment and Children’s Study (JECS) Group

**Affiliations:** 1 Department of Environmental Health, University of Occupational and Environmental Health, Japan, 1-1 Iseigaoka, Yahata-nishi-ku, Kitakyushu, Fukuoka 807-8555, Japan; 2 Department of Obstetrics and Gynecology, University of Occupational and Environmental Health, Japan, 1-1 Iseigaoka, Yahata-nishi-ku, Kitakyushu, Fukuoka 807-8555, Japan; 3 Division of Lifecourse Epidemiology, Integrated Center for Women's Health National Center for Child Health and Development, 2-10-1 Okura, Setagaya-ku, Tokyo 157-8535, Japan; 4 Regional Center for Japan Environment and Children’s Study, University of Occupational and Environmental Health, Japan, 1-1 Iseigaoka, Yahata-nishi-ku, Kitakyushu, Fukuoka 807-8555, Japan; 5 Department of Respiratory Medicine, University of Occupational and Environmental Health, Japan, 1-1 Iseigaoka, Yahata-nishi-ku, Kitakyushu, Fukuoka 807-8555, Japan; 6 Department of Pediatrics, School of Medicine, University of Occupational and Environmental Health, Japan, 1-1 Iseigaoka, Yahata-nishi-ku, Kitakyushu, Fukuoka 807-8555, Japan; 7 Department of Medical Humanities, School of Medicine, University of Occupational and Environmental Health, Japan, 1-1 Iseigaoka, Yahata-nishi-ku, Kitakyushu, Fukuoka 807-8555, Japan; 8 Research Center for Environmental and Developmental Medical Sciences, Kyushu University, 3-1-1 Maidashi, Higashi-ku, Fukuoka 812-8582, Japan; 9 Department of Health Sciences, Graduate School of Medical Sciences, Kyushu University, 3-1-1 Maidashi, Higashi-ku, Fukuoka 812-8582, Japan; 10 Department of Obstetrics and Gynecology, Graduate School of Medical Sciences, Kyushu University, 3-1-1 Maidashi, Higashi-ku, Fukuoka 812-8582, Japan

**Keywords:** Asthma, BMI-SDS, Obesity, Children, The Japan Environment and Children’s Study

## Abstract

**Background:**

The association between obesity and asthma in children has been reported. However, despite its clinical and public health significance, the relationship between longitudinal body mass index standard deviation score (BMI-SDS) trajectories in early life and development of childhood asthma in Japan remains to be elucidated.

**Methods:**

This study used fixed data on 11,433 parent-child pairs from the Japan Environment and Children’s Study, an ongoing national birth cohort study. Information on bronchial asthma was collected from the caregiver-reported questionnaire. The association between body mass index standard deviation score (BMI-SDS) trajectories during the first 3 years of life and the risk of bronchial asthma was examined using multivariate logistic regression analysis.

**Results:**

Children with sustained above-average growth had a higher risk of bronchial asthma at 3 years of age, compared with those with a normative BMI-SDS trajectory (adjusted odds ratio [aOR] 1.31, 95% confidence interval [CI] 1.11–1.55, p = 0.001). At 3 years of age, the association between a persistently high BMI-SDS trajectory and bronchial asthma was more pronounced in boys than in girls (boys: adjusted OR [aOR] 1.39, 95%CI 1.12–1.73, p = 0.003 vs. girls: aOR 1.24, 95%CI 0.96–1.61, p = 0.100). At 4 years of age, similar associations were observed, while the association was stronger in boys, it was significant in girls as well.

**Conclusion:**

The significance of this study is that it captures changes in BMI-SDS over time, rather than at a single point in time, our findings suggest that maintaining a normal BMI-SDS trajectory during childhood may be beneficial for asthma prevention.

**Supplementary information:**

The online version contains supplementary material available at https://doi.org/10.1265/ehpm.26-00162.

## 1. Background

Asthma is one of the most common illnesses among children [1]. The global prevalence, morbidity, and mortality of childhood asthma have increased significantly over the last 40 years [2]. Poor asthma control during childhood can lead to lung growth failure due to repeated airway remodeling. In 2010, Kanno reported that, of 882 adult patients with asthma, 20.4% had childhood-onset asthma, 14.9% had adult recurrent asthma, and approximately 30% had transition or recurrence from childhood asthma [3].

In addition, many studies have shown that obesity increases the severity of asthma; for example, inhaled steroids are less effective in children with obesity-related asthma than in children with non-obesity-related asthma [4]. Parental asthma, prenatal exposure to environmental tobacco smoke, prematurity (particularly very preterm birth), birth by cesarean section, indoor exposure to mold or fungi, and outdoor air pollution are well-established risk factors for childhood asthma [5–8]. In addition, a relationship has been reported between obesity and asthma in children [5, 9, 10]. A cohort study of 5,125 children revealed that rapid weight gain by 3 months of age is a risk factor for asthma symptoms up to 4 years of age, regardless of birth weight [11]. Rzehak et al. [12] also reported that children with rapid body mass index standard deviation score (BMI-SDS) gain in the first 2 years of life had a higher risk of incident asthma up to 6 years of age than children with a less pronounced weight gain slope in early childhood. In contrast, other studies have reported that low, not high, birth weight [13–15], is associated with increased risk of respiratory symptoms or asthma, while, early life weight gain is not [16]. However, despite its importance in clinical and public health, the relationship between longitudinal BMI-SDS trajectories in early life and development of childhood asthma in Japan remains to be elucidated. We believe that examining the association between obesity and bronchial asthma, particularly in early childhood, a critical period of airway development, will facilitate further investigation into the mechanisms of obesity and asthma.

The purpose of this study was to clarify the association between BMI-SDS trajectories during the first 3 years of life and the risk of bronchial asthma at 3 and 4 years of age using a large cohort from the Japan Environment and Children’s Study (JECS).

## 2. Methods

### 2.1. Study design and population

This study was conducted using data from the JECS, a nationwide birth cohort study aimed at investigating the effects of environmental factors on children’s health and development. Detailed protocols have been published elsewhere [17, 18]. The JECS is a birth cohort study of 104,062 fetal records in Japan. The study has been conducted since 2011, with pregnant women recruited between January 2011 and March 2014. The JECS protocol was reviewed and approved by the Ministry of the Environment’s Institutional Review Board on Epidemiological Studies and the Ethics Committees of all participating institutions (Ethical Number: No.100910001). The JECS is conducted in accordance with the principles of the Declaration of Helsinki, and written informed consent was obtained from all participants. The dataset analyzed in the present study was the jecs-ta-20190930 and jecs-qa-20210401 dataset released in October 2019 and April 2021, respectively.

### 2.2. Data collection

Data were primarily collected through self-administered questionnaires and from medical records and the transcriptions of the Maternal and Child Health handbooks. The mothers filled in the questionnaires during the first and second/third trimesters of pregnancy. After birth, the questionnaires at 1 month and every 6 months after birth through the age of 4 years were completed by the guardians. Parental history of asthma and presence of older siblings were assessed in the first trimester. Maternal smoking status was assessed both in the first trimester and at 3 years postpartum. Parents’ educational levels and annual household income were assessed in the second and third trimesters, respectively. Data on childcare facility attendance and breastfeeding status at 1 year of age were collected at 6 months and 1 year of age, respectively. Child’s history of respiratory syncytial virus (RSV) infection was assessed at 6 months and at 1, 1.5, and 2 years of age, while their history of allergic disease was assessed at 1, 1.5, 2, and 3 years of age. Presence of asthma was assessed at 3 and 4 years of age. Data on the children’s sex were transcribed from medical records. Body weight and height at birth were obtained from medical records completed at the delivery institution. At 6 months and 1 year of age, body weight and height were based on measurements recorded in the Maternal and Child Health Handbook during routine health checkups at medical institutions or municipal health centers, which were transcribed into the questionnaires by caregivers. At 1.5, 2, 2.5, and 3 years of age, caregivers reported the most recently measured body weight and height and indicated the location where the measurements had been obtained (e.g., municipal health center, medical institution, nursery school/kindergarten, home, or other).

### 2.3. Exposure

Body mass index standard deviation score (BMI-SDS) trajectories were developed from BMI-SDS that were routinely collected at seven time points during the first 3 years of life (at birth, 6 months, and 1, 1.5, 2, 2.5, and 3 years of age). BMI-SDS were calculated using the Excel-based Clinical Tools for Growth Evaluation of Children, according to the age- and sex-specific reference data from the Japanese Society for Pediatric Endocrinology [19–21].

We used group-based trajectory modeling (GBTM) to identify distinct BMI-SDS trajectory classes and determine the optimal number of classes. The group-based trajectory method presumes that the data comprises of latent distinct groups (trajectories) that best summarize the distinct features as parsimonious as possible [22, 23]. BMI-SDS trajectories were modeled using GBTM with a censored normal distribution. The final number of trajectory groups was not predetermined. Based on previous studies [24], models with 2 to 5 trajectory groups were evaluated, with different polynomial orders considered, resulting in a total of 360 candidate model specifications. For each number of trajectory groups (2–5), the polynomial-order combination with the lowest Bayesian Information Criterion (BIC) was selected as the representative model. The representative models were then compared based on the Akaike Information Criterion (AIC), BIC, entropy, estimated group membership, and average posterior probabilities of group membership. Average posterior probabilities of group membership ≥0.70 were considered indicative of acceptable classification quality according to established criteria [25]. Based on an overall assessment of these indices, the 3-group model (Stata command: order [3 3 3]) was selected as the final model. The 3-group model had an AIC of −104,220.05, a BIC of −104,275.14, and an entropy of 0.777. All three trajectory groups accounted for more than 5% of the study population (22.6%–54.2%), and the average posterior probabilities of group membership ranged from 0.895 to 0.897. The model-fit and classification indices for the 2- to 5-group models are presented in Supplemental Table 1. After selection of the 3-group model, each participant was assigned to the trajectory group for which the posterior probability of membership was highest.

### 2.4. Outcome

Outcome data were collected at 3 and 4 years of age. The incidence of childhood asthma in the JECS was determined using caregiver response to the following question in the International Study of Asthma and Allergies in Childhood (ISAAC) questionnaire for 6–7-year-old children that had been translation-validated in Japanese: “Has your child ever been diagnosed with asthma by a physician?” [26–32]. Children whose caregivers answered “yes” were classified as asthma-positive.

### 2.5. Covariates

Analyses were adjusted for maternal age at delivery, pre-pregnancy BMI, maternal smoking status, parental history of asthma, parental education level, annual household income, child’s history of RSV infection, child’s history of allergic disease, presence of older siblings, childcare facility attendance and breastfeeding status at 1 year of age, with reference to previous studies on the incidence of childhood asthma [8, 33].

### 2.6. Statistical analysis

Three distinct BMI-SDS trajectory classes were identified using GBTM [22, 23] on STATA® Proc Traj. Multiple logistic regression analysis was performed to evaluate the association between group-based trajectory modelling for BMI-SDS trajectories during the first 3 years of life and asthma at 3 years of age. All analyses were performed using STATA version 18 (StataCorp, College Station, TX, USA), with statistical significance set at *p* < 0.05 using a two-tailed test.

## 3. Results

Of 98,412 singleton live births resulting in term-born infants, we excluded those with missing BMI-SDS at zero, 6 months, and 1, 1.5, 2, 2.5, and 3 years of age; missing bronchial asthma data at 3 years of age; and missing covariate data. Finally, data of 11,433 mother–child pairs were analyzed (Fig. 1). Table 1.1 and Table 1.2 show the basic characteristics of the mother–child pairs. Child’s history of RSV infection, child’s history of allergic disease, and maternal history of asthma were more common in the asthma group than in the non-asthma group. The baseline characteristics of included and excluded participants are presented in Supplemental Table 2-1 and Supplemental Table 2-2. At the age of 3 years, 23.2% of the children were classified into Class 1 (average BMI-SDS: −1 to 0), comprising those with persistently lower-than-average growth; 54.2% were classified into Class 2 (average BMI-SDS: 0 to 1), comprising those with a normative BMI-SDS trajectory; and 22.6% were classified into Class 3 (average BMI-SDS: 1 to 1.5), comprising those with sustained above-average growth. BMI-SDSs for each of the Classes are shown in Supplemental Table 3. Children in Class 3 had a higher rate of asthma (9.7%) than those in the other two groups. There are marked differences in BMI SDS between the groups, particularly in the first 1 year of life (Fig. 2, Table 2).

**Fig. 1:**
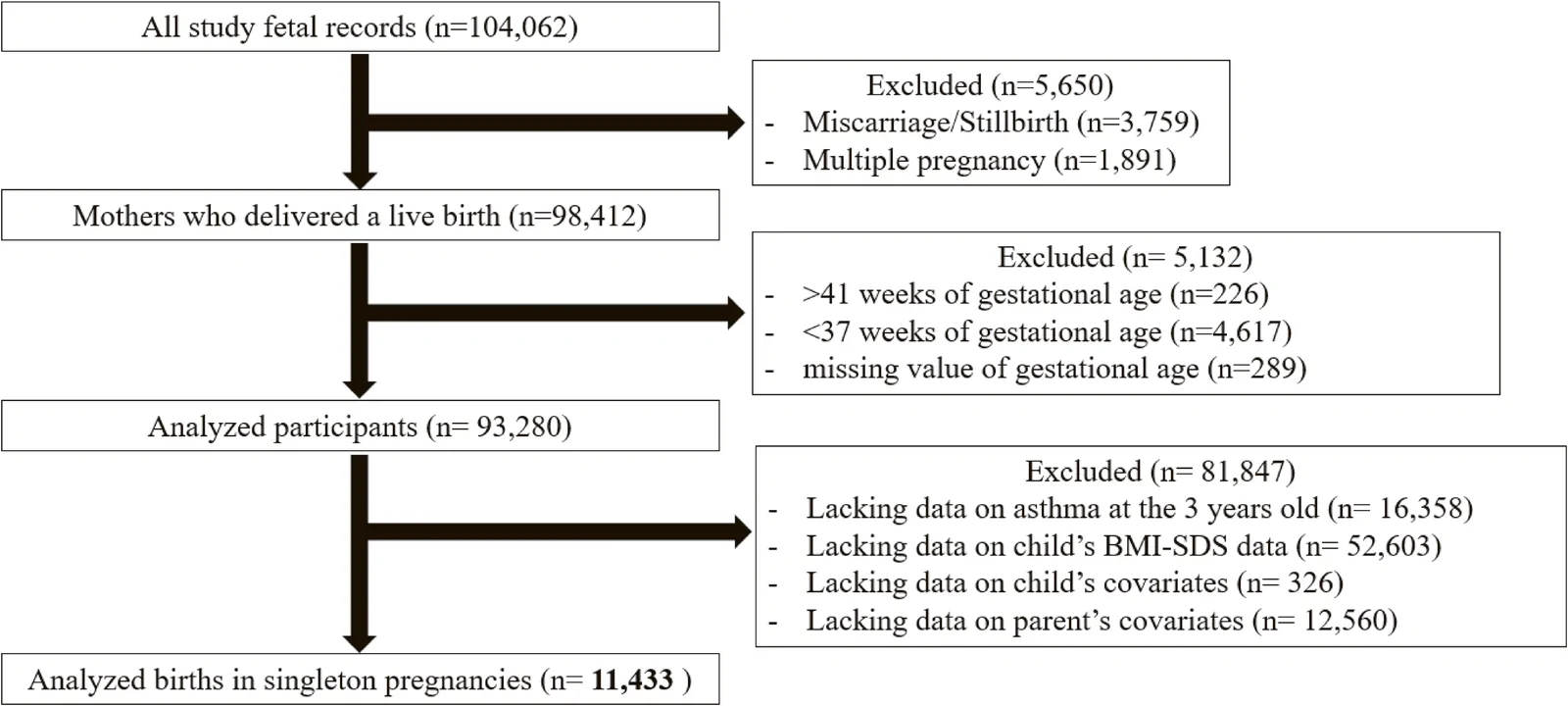
Flow chart of participant selection.

**Table 1.1:** Characteristics of children factors in relation to child’s asthma (n = 11,433).

	**Asthma at the 3-years**		**Asthma at the 4-years**		
	**Negative** **(n = 10,537)**	**Positive** **(n = 896)**	**Negative** **(n = 9,997)**	**Positive** **(n = 984)**	**Missing data** **(n = 452)**
Sex					
Boy	5,237(90.7%)	534(9.3%)	4,973(86.2%)	577(10.0%)	221(3.8%)
Girl	5,300(93.6%)	362(6.4%)	5,024(88.7%)	407(7.2%)	231(4.1%)

History of RSV infection					
Negative	8,564(93.6%)	584(6.4%)	8,104(88.6%)	685(7.5%)	359(3.9%)
Positive	1,973(86.3%)	312(13.7%)	1,893(82.8%)	299(13.1%)	93(4.1%)

Allergic diseases					
No allergy	7,947(94.4%)	474(5.6%)	7,539(89.5%)	542(6.4%)	340(4.1%)
At least one	2,590(86.0%)	422(14.0%)	2,458(81.6%)	442(14.7%)	112(3.7%)

Older siblings					
first-born child	5,618(93.3%)	403(6.7%)	5,313(88.2%)	490(8.1%)	218(3.6%)
second-born child	4,919(90.9%)	493(9.1%)	4,684(86.5%)	494(9.1%)	234(4.4%)

Nursery					
No nursery	7,917(93.2%)	578(6.8%)	7,477(88.0%)	686(8.1%)	332(3.9%)
Nursery	2,620(89.2%)	318(10.8%)	2,520(85.8%)	298(10.1%)	120(4.1%)

Feeding method during the first 6 months postpartum					
Formula	1,628(90.7%)	167(9.3%)	1,553(86.5%)	169(9.4%)	73(4.1%)
Mixed (Formula+breast-feeding)	2,688(91.5%)	249(8.5%)	2,556(87.0%)	257(8.8%)	124(4.2%)
Exclusive breast-feeding	6,221(92.8%)	480(7.2%)	5,888(87.9%)	558(8.3%)	255(3.8%)

RSV: Respiratory syncytial virus

Allergic diseases: Atopic dermatitis, Food allergy, Allergic rhinitis, Allergic conjunctivitis

**Table 1.2:** Characteristics of parents factors in relation to child’s asthma (n = 11,433).

	**Asthma at the 3-years**		**Asthma at the 4-years**		

	**Negative** **(n = 10,537)**	**Positive** **(n = 896)**	**Negative** **(n = 9,997)**	**Positive** **(n = 984)**	**Missing data** **(n = 452)**
Maternal age at delivery, years					
<35	7,867(92.2%)	666(7.8%)	7,469(87.5%)	717(8.4%)	347(4.1%)
≥35	2,670(92.1%)	230(7.9%)	2,528(87.2%)	267(9.2%)	105(3.6%)

Maternal BMI before pregnancy, m^2^/kg					
–18.4	1,564(92.8%)	121(7.2%)	1,488(88.3%)	134(8.0%)	63(3.7%)
18.5–24.9	7,938(92.3%)	666(7.7%)	7,553(87.8%)	731(8.5%)	320(3.7%)
25–	1,035(90.5%)	109(9.5%)	956(83.6%)	119(10.4%)	69(6.0%)

Mother Smoking					
Current Smoker	674(89.2%)	82(10.8%)	622(82.3%)	91(12.0%)	43(5.7%)
Ex-Smoker	3,032(91.8%)	272(8.2%)	2,892(87.5%)	289(8.7%)	123(3.8%)
Non-Smoker	6,831(92.6%)	542(7.4%)	6,483(87.9%)	604(8.2%)	286(3.9%)

Mother’s History of Asthma					
No Asthma	9,523(93.0%)	715(7.0%)	9,041(88.3%)	793(7.7%)	404(4.0%)
Maternal Asthma	1,014(84.9%)	181(15.1%)	956(80.0%)	191(16.0%)	48(4.0%)

Father’s History of Asthma					
No Asthma	9,438(92.7%)	745(7.3%)	8,971(88.1%)	811(8.0%)	401(3.9%)
Paternal Asthma	1,099(88.0%)	151(12.0%)	1,026(82.1%)	173(13.8%)	51(4.1%)

Mother’s years of education, years					
<10	227(88.7%)	29(11.3%)	214(83.6%)	28(10.9%)	14(5.5%)
10–12	2,907(92.4%)	239(7.6%)	2,748(87.3%)	267(8.5%)	131(4.2%)
13–15	4,609(91.7%)	419(8.3%)	4,387(87.3%)	444(8.8%)	197(3.9%)
≥16	2,794(93.0%)	209(7.0%)	2,648(88.2%)	245(8.2%)	110(3.6%)

Father’s years of education, years					
<10	430(90.1%)	47(9.9%)	415(87.0%)	41(8.6%)	21(4.4%)
10–12	3,646(91.4%)	341(8.6%)	3,440(86.3%)	376(9.4%)	171(4.3%)
13–15	2,473(93.1%)	184(6.9%)	2,361(88.9%)	203(7.6%)	93(3.5%)
≥16	3,988(92.5%)	324(7.5%)	3,781(87.7%)	364(8.4%)	167(3.9%)

Household Income, JPY, year					
<2,000,000	389(92.0%)	34(8.0%)	369(87.2%)	37(8.7%)	17(4.1%)
2,000,000–4,000,000	3,405(91.7%)	307(8.3%)	3,240(87.3%)	322(8.7%)	150(4.0%)
4,000,000–6,000,000	3,678(92.3%)	305(7.7%)	3,479(87.3%)	348(8.7%)	156(4.0%)
>6,000,000	3,065(92.5%)	250(7.5%)	2,909(87.8%)	277(8.4%)	129(3.8%)

BMI: Body mass index, ECD1: Junior high school; ECD2: High school; ECD3: Technical junior college, technical/vocational college, or associate degree; ECD4: bachelor’s degree or postgraduate degree.

**Fig. 2:**
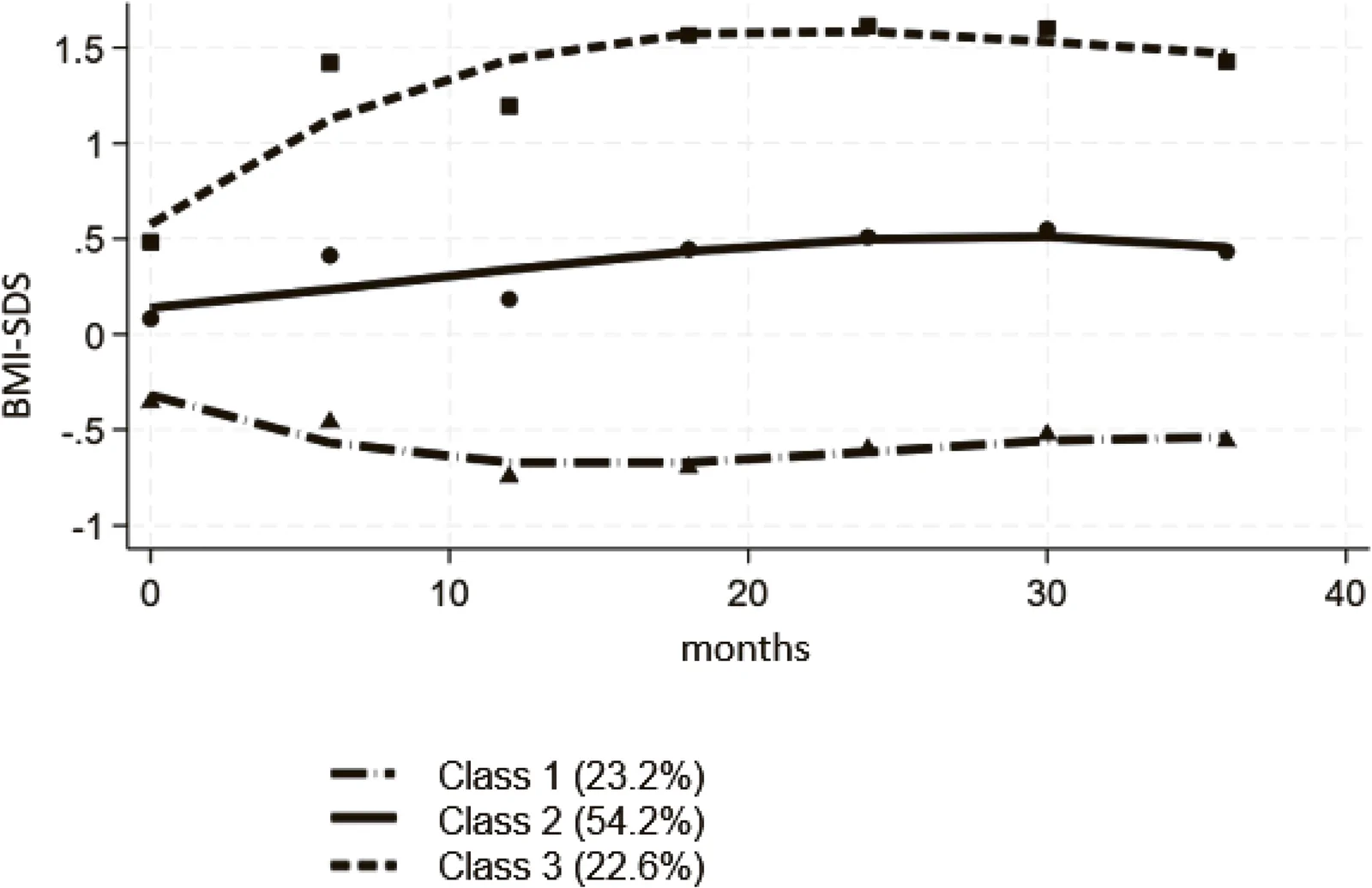
Body mass index trajectory groups to 3 years of age. Percentages shown for each trajectory group represent the model-estimated group membership.

**Table 2:** BMI-SDS trajectory group to 3 years old in relation to child’s asthma (n = 11,433).

		**Asthma at the 3-years**		**Asthma at the 4-years**		
		**Negative** **(n = 10,537)**	**Positive** **(n = 896)**	**Negative** **(n = 9,997)**	**Positive** **(n = 984)**	**Missing data** **(n = 452)**
BMI-SDS trajectory group	Class 1	2,413(93.2%)	175(6.8%)	2,296(88.7%)	186(7.2%)	106(4.1%)
	Class 2	5,834(92.5%)	475(7.5%)	5,538(87.8%)	531(8.4%)	240(3.8%)
	Class 3	2,290(90.3%)	246(9.7%)	2,163(85.3%)	267(10.5%)	106(4.2%)

Regarding asthma incidence at 3 years of age, univariate analysis showed that children with sustained above-average growth had a higher risk of bronchial asthma than those with a normative BMI-SDS trajectory (crude odds ratio [cOR] 1.32, 95%CI 1.12–1.55, *p* = 0.001) (Table 3.1). The same trend was observed in the multivariate regression analysis (adjusted odds ratio [aOR] 1.31, 95% CI 1.11–1.55, *p* = 0.001); this trend was significant in boys (aOR 1.39, 95%CI 1.12–1.73, *p* = 0.003), not in girls (aOR 1.24, 95%CI 0.96–1.61, *p* = 0.100) (Table 3.2).

**Table 3.1:** BMI-SDS trajectory group in relation to child’s asthma at the 3-years (Crude models).

	**Total**		
	**cOR**	**95% CI**	**P-value**
BMI-SDS trajectory group			
Class 1	0.89	0.74–1.07	0.207
Class 2	1(Referent)		
Class 3	1.32	1.12–1.55	0.001

**Table 3.2:** BMI-SDS trajectory group in relation to child’s asthma at the 3-years (adjusted models).

	**Total**			**Boys**			**Girls**		
	**aOR**	**95% CI**	**P-value**	**aOR**	**95% CI**	**P-value**	**aOR**	**95% CI**	**P-value**
BMI-SDS trajectory group									
Class 1	0.92	0.76–1.11	0.376	0.86	0.68–1.09	0.218	0.98	0.73–1.32	0.917
Class 2	1(Referent)			1(Referent)			1(Referent)		
Class 3	1.31	1.11–1.55	0.001	1.39	1.12–1.73	0.003	1.24	0.96–1.61	0.100

cOR, crude odds ratio; aOR, adjusted odds ratio; CI, confidence interval.

The following confounders were included in the multivariate analysis: maternal age at delivery, pre-pregnancy BMI, maternal smoking status, parental history of asthma, parental education level, annual household income, child’s history of RSV infection, child’s history of allergic disease, presence of older siblings, childcare facility attendance and breastfeeding status at 1 year of age.

Regarding asthma incidence at 4 years of age, univariate analysis showed that children with sustained above-average growth had a higher risk of bronchial asthma than those with a normative BMI-SDS trajectory (cOR 1.29, 95%CI 1.10–1.50, *p* = 0.001) (Table 4.1). Multivariate analysis showed that children with sustained above-average growth had a higher risk of bronchial asthma than those with a normative BMI-SDS trajectory (aOR 1.29, 95%CI 1.10–1.52, *p* = 0.002); this trend was significant in boys (aOR 1.29, 95%CI 1.04–1.60, *p* = 0.019), in girls (aOR 1.32, 95%CI 1.03–1.67, *p* = 0.025) (Table 4.2).

**Table 4.1:** BMI-SDS trajectory group in relation to child’s asthma at the 4-years (Crude models).

	**Total**		
	**cOR**	**95% CI**	**P-value**
BMI-SDS trajectory group			
Class 1	0.84	0.71–1.01	0.058
Class 2	1(Referent)		
Class 3	1.29	1.10–1.50	0.001

**Table 4.2:** BMI-SDS trajectory group in relation to child’s asthma at the 4-years (adjusted models).

	**Total**			**Boys**			**Girls**		
	**aOR**	**95% CI**	**P-value**	**aOR**	**95% CI**	**P-value**	**aOR**	**95% CI**	**P-value**
BMI-SDS trajectory group									
Class 1	0.85	0.71–1.02	0.074	0.90	0.72–1.13	0.361	0.75	0.55–1.00	0.052
Class 2	1(Referent)			1(Referent)			1(Referent)		
Class 3	1.29	1.10–1.52	0.002	1.29	1.04–1.60	0.019	1.32	1.03–1.67	0.025

cOR, crude odds ratio; aOR, adjusted odds ratio; CI, confidence interval.

The following confounders were included in the multivariate analysis: maternal age at delivery, pre-pregnancy BMI, maternal smoking status, parental history of asthma, parental education level, annual household income, child’s history of RSV infection, child’s history of allergic disease, presence of older siblings, childcare facility attendance and breastfeeding status at 1 year of age.

## 4. Discussion

In this large-scale prospective cohort study conducted using data obtained across Japan, we found that sustained above-average BMI-SDS trajectories during the first three years of life were significantly associated with asthma at 3 and 4 years of age compared with normative BMI-SDS trajectories. Furthermore, this association between persistently high BMI-SDS trajectories and asthma appeared to be more pronounced in boys than in girls. Nonetheless, a significant association was also observed in girls at 4 years of age, suggesting that persistently high BMI-SDS may be associated with increased asthma risk in both sexes, although the magnitude of the association differs between boys and girls.

To the best of our knowledge, this is the first report showing a longitudinal association between early childhood BMI-SDS trajectories and the risk of bronchial asthma in a large-scale prospective cohort study in Japan. Based on the results of our study, the following three mechanisms may be responsible for the relationship between sustained above-average growth in children, especially boys, and the development of asthma [34].

The mechanism is mechanical impediment. Obesity reduces respiratory system compliance, lung volume, and airway caliber. Notably, the persistently high BMI-SDS trajectory in our study does not necessarily represent clinical obesity, and sustained excess body weight may induce similar physiological changes. Forno et al. reported that obesity in children might be caused by obstructive ventilatory impairment due to dysanapsis [35]. Dysanapsis was coined by Green et al., and it describes a disproportionate increase in lung parenchymal volume relative to airway diameter, and obese children experience this maladjusted growth [36]. This is because the intraparenchymal airway diameter was entirely dependent on lung recoil pressure and relatively independent of lung volume [37]. In particular, the risk of dysanapsis from obesity was higher in boys [35]. Specifically, obesity is associated with lung growth, leading to incongruence between the growth of the lung parenchyma and airway caliber, which may play a critical role in the airflow limitation observed in children with obesity.

In addition, higher workload from adipose tissue deposition in the abdomen and chest wall of individuals with obesity and overweight forces them to breathe at lower tidal volumes, reducing airway smooth muscle cell stretching, and increasing airway resistance over time [38]. These phenomena induce, bronchial remodeling over time, followed by airway hyperreactivity and a consequent lung volume reduction, increasing the risk of developing bronchial asthma [39].

The second mechanism is adipokine production. Obesity is associated with higher circulating levels of leptin and resistin as well as a reduction in circulating adiponectin [40]. Leptin production is proportionate to the adipocyte mass, functioning as a proinflammatory hormone inducing neutrophil chemotaxis, active oxygen species production, natural killer cell and macrophage activation, as well as the production of Th1 cytokines, including interleukin (IL)-6 and interferon-γ [41]. Higher leptin levels appear to be associated with increased risk of asthma development in children, particularly in boys with atopy [42].

The third and final mechanism is hypoxic inflammation. Rapid and excessive accumulation of adipose tissue generates a hypoxic microenvironment that promotes the differentiation of circulating monocytes into patrolling monocytes, which in turn secrete inflammatory cytokines, including monocyte chemoattractant protein-1, tumor necrosis factor, and IP-10, cytokines of the Th1 immune profile26. Furthermore, obesity worsened IL-6 production, which is typical of the inflammatory background of asthma [43].

These mechanisms may explain why persistent obesity or sustained overweight during early childhood may contribute to the development of childhood asthma. The persistently high BMI-SDS trajectory identified in our study is more likely to reflect persistent overweight rather than clinical obesity. Therefore, our findings suggest that, in addition to preventing obesity, preventing excessive weight gain during early childhood may be important for reducing the risk of childhood asthma. Early childhood represents a critical period for establishing lifelong health behaviors, and interventions initiated during the preschool years have been shown to be effective in preventing overweight and obesity [44]. Furthermore, previous studies have demonstrated that children with obesity tend to have greater asthma severity [45–47], poorer disease control [48], and lower quality of life [49] than their normal-weight peers. Collectively, these findings suggest that preventing excessive weight gain during early childhood may not only reduce the risk of childhood asthma but also help mitigate adverse asthma-related outcomes later in life.

## 5. Limitations

Our study has some limitations. First, the assessment of body weight and height varied across follow-up visits. Specifically, at 6 months and 1 year of age, body weight and height were primarily measured during routine health checkups and transcribed from the Maternal and Child Health Handbooks by caregivers. In contrast, at 1.5, 2, 2.5, and 3 years of age, caregivers reported their child’s most recently measured body weight and height. These differences in data collection methods may have introduced transcription and reporting errors, as well as variability in measurement settings, potentially resulting in non-differential misclassification of BMI-SDS.

Second, physician-diagnosed asthma was assessed using caregiver-completed questionnaires rather than using confirmed physician’s diagnosis through medical records or other clinical documentation; this may have likely introduced underreporting and recall bias. However, the questionnaire item used to assess asthma was derived from the ISAAC, a validated instrument that has been widely used to identify childhood asthma in international epidemiological studies.

Third, it was difficult to confirm asthma diagnosis in infants and preschool children [50], and some outcomes may have been misclassified. For example, a study showed that 60% of children who experienced wheezing before 3 years of age stopped wheezing by the age of 6 years [24, 51], and we assessed the presence of asthma at ages 3 and 4 years, which may not have assessed actual asthma. Furthermore, because the present analysis was constrained by the availability of the JECS fixed dataset, asthma outcomes of the participants could only be evaluated for up to 4 years of age. To address this limitation, we performed an additional multivariable logistic regression analysis to examine the association between BMI-SDS trajectories during the first 3 years of life and wheezing phenotypes in children before the age of 3 years [52] (namely, never, early transient, late-onset, and persistent wheezing). The multivariate analysis showed that children with sustained above-average growth had a higher risk of persistent wheezing at 3 years of age than those with a normative BMI-SDS trajectory (Supplemental Table 4-1 and Supplemental Table 4-2). The presence of wheezing episodes was assessed using a self-administered questionnaire completed by the mothers or caregivers when their children were 1 and 3 years old. Based on the questionnaires of the ISAAC, current wheeze was defined as a positive response to the following question: “Has your child ever had wheezing or whistling in the chest in the last 12 months?” [29, 31, 53]. The children were assigned to the following four groups according to their history of wheezing with reference to the study by Martinez et al. [51]: 1) never wheezing, 2) early transient wheezing, 3) late-onset wheezing, and 4) persistent wheezing. Future studies using longer-term follow-up data are needed to validate our findings regarding the association between BMI-SDS trajectories and childhood asthma.

Fourth, we assumed that obesity precedes asthma, but the possibility of reverse causation remains. Clarifying the temporal relationship between adiposity and subsequent asthma outcomes remains a complex and unresolved challenge in the field [24].

Fifth, because participants with missing data regarding exposure, outcomes, or covariates were excluded from the analyses, selection bias may have been introduced. Although there were significant differences regarding the baseline characteristics of the included and excluded participants, the standardized mean difference (SMD) were generally small (all absolute SMDs <0.20), suggesting that the observed differences were limited despite the large sample size. However, because this study was based on a complete-case analysis, the generalizability of our findings to the original JECS cohort should be interpreted with caution.

## 6. Conclusion

In conclusion, this large-scale prospective cohort study of 11,433 infants revealed that children with sustained above-average growth during the first three years of life had a higher risk of bronchial asthma than those with a normative BMI-SDS trajectory. The significance of this study is that it captures changes in BMI-SDS over time, rather than at a single point in time. Our data should facilitate further investigations of mechanisms underlying the association between obesity and non-allergic asthma in early childhood, which is a critical period of airway development. These results might facilitate the early identification of children at highest risk for asthma, as well as opportunities for early intervention measures, including — nutritional guidance, promotion of physical activity, and sleep optimization [54, 55], for obesity-related factors, to prevent asthma in this high-risk population. This finding suggests that maintaining a normal BMI-SDS trajectory during childhood may be beneficial for preventing asthma in Japanese children.

## Abbreviations

AIC: Akaike Information Criterion
aOR: adjusted odds ratio
BIC: Bayesian Information Criterion
BMI: body mass index
BMI-SDS: body mass index standard deviation score
CI: confidence interval
cOR: crude odds ratio
GBTM: group-based trajectory modeling
ISAAC: International Study of Asthma and Allergies in Childhood
JECS: Japan Environment and Children’s Study
OR: odds ratio
RSV: respiratory syncytial virus
SMD: standardized mean difference
OR: odds ratio

## Supplementary information

**Additional file 1:** **Supplemental Table 1(in Methods).** Comparison of model fit statistics for the selected trajectory models with two to five trajectory groups. **Supplemental Table 2-1(in Results).** Baseline characteristics of children factors among included and excluded participants. **Supplemental Table 2-2(in Results).** Baseline characteristics of parental factors among included and excluded participants. **Supplemental Table 3(in Results).** BMI value for each BMI-SDS trajectory group to 3 years old (n = 11,433). **Supplemental Table 4-1(in Limitation).** BMI-SDS trajectory group to 3 years old in relation to wheezing phenotypes (n = 11,433). **Supplementary Table 4-2(in Limitation).** Associations between BMI-SDS trajectory group and wheezing phenotypes (adjusted models).

## Declarations

### Appendix

Members of the JECS Group as of 2025: Michihiro Kamijima (Principal Investigator, Nagoya City University, Nagoya, Japan), Shin Yamazaki (National Institute for Environmental Studies, Tsukuba, Japan), Maki Fukami (National Center for Child Health and Development, Tokyo, Japan), Reiko Kishi (Hokkaido University, Sapporo, Japan), Chiharu Ota (Tohoku University, Sendai, Japan), Koichi Hashimoto (Fukushima Medical University, Fukushima, Japan), Kenichi Sakurai (Chiba University, Chiba, Japan), Shuichi Ito (Yokohama City University, Yokohama, Japan), Ryoji Shinohara (University of Yamanashi, Chuo, Japan), Hidekuni Inadera (University of Toyama, Toyama, Japan), Takeo Nakayama (Kyoto University, Kyoto, Japan), Ryo Kawasaki (Osaka University, Suita, Japan), Yasuhiro Takeshima (Hyogo Medical University, Nishinomiya, Japan), Hideki Nagashima (Tottori University, Yonago, Japan), Narufumi Suganuma (Kochi University, Nankoku, Japan), Mayumi Tsuji (University of Occupational and Environmental Health, Kitakyushu, Japan), and Kimitoshi Nakamura (Kumamoto University, Kumamoto, Japan).
