# Supplementary material for: Relationship between BMI-SDS trajectories and childhood asthma: a prospective cohort study from the Japan Environment and Children’s Study
Source: Environ Health Prev Med. 2026 Sep 29;31:65. doi: 10.1265/ehpm.26-00162 (PMC13649615; doi:10.1265/ehpm.26-00162)
Supplement: Supplementary file 1 [file ehpm-31-065-s001.docx]

**Supplemental Table 1(in Methods).** Comparison of model fit statistics for the selected trajectory models with two to five trajectory groups.

| Number of trajectory groups | Polynomial order | AIC | BIC | Entropy | Trajectory class | Estimated group membership (%) | Average posterior probability of group membership |
| --- | --- | --- | --- | --- | --- | --- | --- |
| 2 | (3, 3) | -104,219.87 | -104,275.60 | 0.727 | Class 1 | 51.9% | 0.911 |
|  |  |  |  |  | Class 2 | 48.1% | 0.929 |
| 3 | (3, 3, 3) | -104,220.05 | -104,275.14 | 0.777 | Class 1 | 23.2% | 0.895 |
|  |  |  |  |  | Class 2 | 54.2% | 0.897 |
|  |  |  |  |  | Class 3 | 22.6% | 0.897 |
| 4 | (3, 3, 1, 1) | -103,705.27 | -103,764.02 | 0.777 | Class 1 | 15.1% | 0.879 |
|  |  |  |  |  | Class 2 | 33.4% | 0.867 |
|  |  |  |  |  | Class 3 | 46.8% | 0.868 |
|  |  |  |  |  | Class 4 | 4.7% | 0.860 |
| 5 | (3, 3, 2, 1, 3) | -103,095.12 | -103,175.91 | 0.698 | Class 1 | 11.8% | 0.871 |
|  |  |  |  |  | Class 2 | 22.6% | 0.726 |
|  |  |  |  |  | Class 3 | 41.0% | 0.845 |
|  |  |  |  |  | Class 4 | 15.7% | 0.686 |
|  |  |  |  |  | Class 5 | 8.9% | 0.847 |

AIC: Akaike Information Criterion, BIC: Bayesian Information Criterion

For each number of trajectory groups (2–5), candidate models with different polynomial-order combinations were systematically evaluated, and the polynomial-order combination with the lowest BIC was selected as the representative model for that group number. Accordingly, (3, 3), (3, 3, 3), (3, 3, 1, 1), and (3, 3, 2, 1, 3) were selected for the 2-, 3-, 4-, and 5-group models, respectively. The representative models were then compared based on model fit (AIC and BIC), classification quality (entropy and average posterior probabilities of group membership), and estimated group membership.

**Supplemental Table 2-1(in Results).**

Baseline characteristics of children factors among included and excluded participants.

|  | Included | Excluded | Total | P-value | SMD |
| --- | --- | --- | --- | --- | --- |
| Sex |  |  |  |  |  |
| Boy | 5,771 | 41,731 | 47,502 | 0.515 | 0.010 |
| Girl | 5,662 | 40,107 | 45,769 |  |  |
| History of RSV infection |  |  |  |  |  |
| Negative | 9,148 | 53,021 | 62,169 | 0.003 | 0.030 |
| Positive | 2,285 | 14,271(%) | 16,556 |  |  |
| Allergic diseases |  |  |  |  |  |
| No allergy | 8,421 | 45,738 | 54,159 | <0.001 | 0.057 |
| At least one | 3,012 | 18,564 | 21,576 |  |  |
| Older siblings |  |  |  |  |  |
| first-born child | 6,021 | 35,306 | 41,327 | <0.001 | 0.180 |
| second-born child | 5,412 | 45,398 | 50,810 |  |  |
| Nursery |  |  |  |  |  |
| No nursery | 8,495 | 52,883 | 61,378 | <0.001 | 0.033 |
| Nursery | 2,938 | 19,726 | 22,664 |  |  |
| Feeding method during the first 6 months postpartum | | | | | |
| Formula | 1,795 | 13,988 | 15,783 | <0.001 | 0.063 |
| Mixed  (Formula+breast-feeding) | 2,937 | 16,810 | 19,747 |  |  |
| Exclusive breast-feeding | 6,701 | 41,440 | 48,141 |  |  |

SMD: standardized mean difference

RSV: Respiratory syncytial virus

Allergic diseases：Atopic dermatitis, Food allergy, Allergic rhinitis, Allergic conjunctivitis

Data are presented as n (%). Percentages were calculated among participants with available data for each variable. Totals may not equal the overall sample size because of missing values.

**Supplemental Table 2-2(in Results).**

Baseline characteristics of parental factors among included and excluded participants.

|  | Included | Excluded | Total | P-value | SMD |
| --- | --- | --- | --- | --- | --- |
| Maternal age at delivery, years |  |  |  |  |  |
| <35 | 8,533 | 61,235 | 69,769 | 0.663 | 0.004 |
| ≥ 35 | 2,900 | 20,612 | 23,512 |  |  |
| Maternal BMI before pregnancy, m^2^/kg | | | | |  |
| –18.4 | 1,685 | 12,766 | 14,451 | <0.001 | 0.003 |
| 18.5–24.9 | 8,604 | 60,066 | 68,670 |  |  |
| 25– | 1,144 | 9,015 | 10,159 |  |  |
| Mother Smoking |  |  |  |  |  |
| Current Smoker | 756 | 6,341 | 7,097 | <0.001 | 0.132 |
| Ex-Smoker | 3,304 | 19,662 | 22,966 |  |  |
| Non-Smoker | 7,373 | 37,887 | 45,260 |  |  |
| Mother's History of Asthma |  |  |  |  |  |
| No Asthma | 10,238 | 71,812 | 82,050 | 0.069 | 0.018 |
| Maternal Asthma | 1,195 | 8,892 | 10,087 |  |  |
| Father's History of Asthma |  |  |  |  |  |
| No Asthma | 10,183 | 31,833 | 42,016 | 0.788 | 0.003 |
| Paternal Asthma | 1,250 | 3,871 | 5,121 |  |  |
| Mother's years of education, years | | | | | |
| <10 | 256 | 4,139 | 4,395 | <0.001 | 0.150 |
| 10-12 | 3,146 | 25,581 | 28,727 |  |  |
| 13-15 | 5,028 | 33,368 | 38,396 |  |  |
| ≥16 | 3,003 | 16,828 | 19,831 |  |  |
| Father's years of education, years | | | | | |
| <10 | 477 | 6,142 | 6,619 | <0.001 | 0.100 |
| 10-12 | 3,987 | 29,385 | 33,372 |  |  |
| 13-15 | 2,657 | 17,789 | 20,446 |  |  |
| ≥16 | 4,312 | 26,016 | 30,328 |  |  |
| Household Income, JPY, year |  |  |  |  |  |
| <2,000,000 | 423 | 4,375 | 4,798 | <0.001 | 0.006 |
| 2,000,000- 4,000,000 | 3,712 | 25,782 | 29,494 |  |  |
| 4,000,000- 6,000,000 | 3,983 | 24,221 | 28,204 |  |  |
| >6,000,000 | 3,315 | 19,506 | 22,821 |  |  |

SMD: standardized mean difference, BMI: Body mass index, ECD1: Junior high school; ECD2: High school; ECD3: Technical junior college, technical/vocational college, or associate degree; ECD4: bachelor’s degree or postgraduate degree.

Data are presented as n (%). Percentages were calculated among participants with available data for each variable. Totals may not equal the overall sample size because of missing values.

**Supplemental Table 3(in Results)**. BMI value for each BMI-SDS trajectory group to 3 years old（n=11,433）.

|  |  | BMI (kg/m^2^), mean, SD | | | | | | |
| --- | --- | --- | --- | --- | --- | --- | --- | --- |
|  |  | birth | 6 months | 1 year | 1.5 years | 2 years | 2.5 years | 3 years |
| BMI-SDS trajectory group | Class 1 | 12.19±1.04  (8.3-16.49) | 15.94±1.08  (10.82-20.38) | 15.72±0.83  (12.26-18.69) | 15.17±1.02  (9.53-22.05) | 15.01±0.86  (9.79-19.19) | 14.93±0.82  (10.63-18.59) | 14.78±0.82  (10-23.04) |
|  | Class 2 | 12.70±1.04  (8.68-24.62) | 17.15±1.16  (10.47-21.5) | 17.01±0.92  (13.64-20.83) | 16.63±0.98  (10.96-21.51) | 16.38±0.87  (12.48-22.99) | 16.25±0.90  (12.19-25.91) | 15.99±0.84  (10.93-24.93) |
|  | Class 3 | 13.18±1.10  (9.84-29.52) | 18.62±1.40  (10.75-23.77) | 18.51±1.12  (15.25-24.75) | 18.22±1.21  (12.62-27.71) | 17.92±1.07  (14.27-29.84) | 17.72±1.05  (13.8-24.89) | 17.39±1.00  (14.24-24.94) |

**Supplemental Table 4-1(in Limitation).** BMI-SDS trajectory group to 3 years old in relation to wheezing phenotypes（n=11,433）.

|  |  | Early transient wheezing | Late-onset  wheezing | Persistent  wheezing | Never wheezing | missing data |
| --- | --- | --- | --- | --- | --- | --- |
| BMI-SDS trajectory group | Class 1 | 132(5.1%) | 239(9.2%) | 145(5.6%) | 356(13.8%) | 1,716(66.3%) |
|  | Class 2 | 376(6.0%) | 688(10.9%) | 404(6.4%) | 917(14.5%) | 3,924(62.2%) |
|  | Class 3 | 219(8.6%) | 295(11.6%) | 217(8.6%) | 373(14.7%) | 1,432(56.5%) |

1) never wheezing (those with no wheezing by 2–3 years of age); 2) early transient wheezing (those with wheezing by 1 year of age but no wheezing by 2–3 years of age); 3) late-onset wheezing(those with no wheezing by 1 year of age, but with wheezing by 2–3 years of age); and 4) persistent wheezing (those with wheezing both by 1 and 2–3 years of age).

**Supplementary Table 4-2(in Limitation).** Associations between BMI-SDS trajectory group and wheezing phenotypes (adjusted models).

|  | Early transient wheezing | | |  | Late-onset wheezing | | |  | Persistent wheezing | | |
| --- | --- | --- | --- | --- | --- | --- | --- | --- | --- | --- | --- |
|  | aOR | 95% CI | P-value |  | aOR | 95% CI | P-value |  | aOR | 95% CI | P-value |
| BMI-SDS trajectory group |  |  |  |  |  |  |  |  |  |  |  |
| Class 1 | 0.95 | 0.74-1.22 | 0.667 |  | 0.88 | 0.73-1.07 | 0.211 |  | 1.01 | 0.79-1.29 | 0.930 |
| Class 2 | 1(Referent) |  |  |  | 1(Referent) |  |  |  | 1(Referent) |  |  |
| Class 3 | 1.38 | 1.10-1.72 | 0.005 |  | 1.08 | 0.90-1.30 | 0.405 |  | 1.30 | 1.03-1.62 | 0.024 |

cOR, crude odds ratio; aOR, adjusted odds ratio; CI, confidence interval.

Adjusted for maternal age at delivery, pre-pregnancy BMI, maternal smoking status, parental history of asthma, parental education level, annual household income, child’s history of RSV infection, child’s history of allergic disease, presence of older siblings, childcare facility attendance and breastfeeding status at 1 year of age.
